# A Review of Catfish Genomics: Progress and Perspectives

**DOI:** 10.1002/cfg.265

**Published:** 2003-04

**Authors:** Zhanjiang Liu

**Affiliations:** The Fish Molecular Genetics and Biotechnology Laboratory, 203 Swingle Hall Department of Fisheries and Allied Aquacultures and Program of Cell and Molecular Biosciences Aquatic Genomics Unit Auburn University Auburn AL 36849 USA

## Abstract

Catfish is one of the lower teleosts whose genome research is important for evolutionary genomics. As the major aquaculture species in the USA, its genome research also has practical and economical implications. Much progress has been made in recent years, including the development of large numbers of molecular markers, the construction of framework genetic linkage maps, the identification of putative markers involved in performance traits, and the development of genomic resources. Repetitive elements have been identified and characterized in the catfish genome that should facilitate physical analysis of the catfish genome. A large number of genes or full-length cDNAs have been analysed using genomic approaches, providing information on gene structure, gene evolution and gene expression in relation to functions. Catfish genome research has come to a stage when physical mapping through BAC contig construction is greatly demanded, in order to develop regional markers for QTL analysis and for large-scale comparative mapping. The current effort in large-scale EST analysis and type I marker mapping should further enhance research efficiency through comparative mapping. Candidate gene identification is being accelerated through the use of cDNA microarrays.


**Comparative and Functional Genomics**
*Comp Funct Genom* 2003; **4**: 259–265.
Published online 1 April 2003 in Wiley InterScience (www.interscience.wiley.com). **DOI:** 10.1002/cfg.265

**Conference Review**

### A review of catfish genomics: progress and perspectives

Zhanjiang Liu*
*The Fish Molecular Genetics and Biotechnology Laboratory, Department of Fisheries and Allied Aquacultures and Program of Cell and Molecular Biosciences, Aquatic Genomics Unit, Auburn University, Auburn, AL 36849, USA*

**Correspondence to:*
*Zhanjiang Liu, 203 Swingle Hall, Department of Fisheries, Auburn University, Auburn, AL 36849, USA.*
*E-mail: zliu@acesag.auburn.edu*

Received: 21 January 2003
Revised: 31 January 2003
Accepted: 31 January 2003

### Abstract

**Catfish is one of the lower teleosts whose genome research is important for evolutionary genomics. As the major aquaculture species in the USA, its genome research also has practical and economical implications. Much progress has been made in recent years, including the development of large numbers of molecular markers, the construction of framework genetic linkage maps, the identification of putative markers involved in performance traits, and the development of genomic resources. Repetitive elements have been identified and characterized in the catfish genome that should facilitate physical analysis of the catfish genome. A large number of genes or full-length cDNAs have been analysed using genomic approaches, providing information on gene structure, gene evolution and gene expression in relation to functions. Catfish genome research has come to a stage when physical mapping through BAC contig construction is greatly demanded, in order to develop regional markers for QTL analysis and for large-scale comparative mapping. The current effort in large-scale EST analysis and type I marker mapping should further enhance research efficiency through comparative mapping. Candidate gene identification is being accelerated through the use of cDNA microarrays. Copyright © 2003 John Wiley & Sons, Ltd.**

**Keywords:** **fish; catfish; QTL; EST; BAC; genomics; traits; marker; microarray; linkage map**

Teleosts are a diverse group of more than 23 000 species, by far the largest group of vertebrates. They offer unique systems for genomic studies. In spite of high levels of biological conservation in both genomes and functions, the extent to which teleosts can adapt to environment (e.g. low oxygen, high pressure, or a wide range of temperatures) is well beyond imagination for any higher vertebrates, such as mammals. Therefore, genomic studies using an aquaculture fish species provide unique scientific information concerning genetic mechanisms governing performance traits in aquatic environments in relation to genome evolution, on top of their economic significance. The catfish is an excellent model organism for genomic studies, particularly for agriculturally important issues. Its high fecundity allows production of large families, with thousands of progeny. This would offer a great opportunity for QTL scans, with a uniform genetic background allowing extensive phenotype selection using selective genotyping, thereby reducing time, money and efforts in QTL analysis.

Catfish genome mapping was initiated in the early 1990s [12,29]. However, the lack of polymorphic molecular markers prohibited its rapid progress. Large-scale catfish genome research is only a recent event, following the first Aquaculture Genome Workshop, held in 1997. This review will cover recent progress made in molecular marker development, linkage mapping, QTL analysis, development of genomic resources, characterization of genomic composition, and characterization of genes in specific pathways.

Copyright © 2003 John Wiley & Sons, Ltd.

### Development and evaluation of molecular markers

The initial effort in catfish genomics was devoted to the development of polymorphic markers and the evaluation of their applications in catfish. At the beginning, marker systems requiring no prior molecular information were evaluated. In this regard, random amplified polymorphic DNA (RAPD) and amplified fragment length polymorphism (AFLP) markers do not require any probes or sequence information. They are applicable to species where there is no prior molecular biological information, such as catfish. RAPD polymorphism was high using the channel catfish × blue catfish hybrid system, but was relatively low among channel catfish [18]. A total of 142 primers were tested for their application in genetic studies of catfish [19]. RAPD markers should be highly useful for hybrid identification in catfish, but are not most suitable for genome mapping because of their relatively low reproducibility, due to the use of low-temperature PCR. AFLPs are similar to RAPDs in their inheritance as dominant markers [25]. However, several features of AFLP make it one of the most preferable markers in catfish. First, it is a highly robust marker system, allowing multiple loci to be simultaneously analysed. Second, the polymorphism rate is high, especially for analysis using the channel catfish × blue catfish hybrid resource families. In contrast to the low reproducibility of RAPD, AFLP is highly reliable. Over 3000 polymorphic AFLPs were identified, using 64 primer combinations [23]. AFLP markers were not only very useful for genome mapping in the catfish [14], but also highly useful for population studies [28].

As progress was made in catfish genomics, microsatellite markers were required. Several hundred microsatellite markers have been developed in catfish [5,11,21,26,27,33,35]. Microsatellite markers are very useful markers. Their major strengths lie in their high polymorphism, co-dominant inheritance, high abundance, even distribution in the genome, and small locus size facilitating genotyping using PCR. Their major drawback is the cost and effort involved in the development of the markers. Like RAPD and AFLP, most microsatellites are type II markers that do not allow information communication among different species through the evolutionary spectrum.

### Type I markers

One of the lessons learned from the initial efforts in catfish genomics was that not enough attention was paid to the development of type I markers. Like type II markers, type I markers are useful for genetic linkage and QTL mapping. However, the additional benefits of being able to conduct comparative genome mapping, to study genome evolution, to allow inter-species information exchange and to enhance inter-laboratory communications can only be offered by type I markers. Three approaches have been taken to develop type I markers in catfish. The first approach was to identify microsatellites within cDNAs [21,27]. While genes offer functional sequences, microsatellites offer highly polymorphic sequences. Polymorphic microsatellites within genes of known function would convert the microsatellites into type I markers. It appears that catfish ESTs are rich in microsatellites. About 9% of catfish ESTs deposited to GenBank contain microsatellites, i.e. twice the rate of zebrafish, and seven times of the rate in mammals. The second approach was to identify single nucleotide polymorphisms (SNPs) among expressed sequences to find expressed single nucleotide polymorphisms (eSNPs). In this effort, we have taken advantage of the channel catfish × blue catfish interspecific hybrid system. Comparative analysis of expressed sequence tags (ESTs) has proved to be a very effective way to develop type I SNPs. To date, a total of 86 603 bases have been analysed from 159 genes, of which 63 537 bases were analysed from 131 known genes. Among the 131 known genes, a total of 840 eSNPs were identified, i.e. 1.32 eSNPs per 100 bp of known genes. The vast majority of the genes harbour at least one SNP between channel catfish and blue catfish. The third approach is to identify microsatellites within introns. Catfish introns are rich in microsatellite sequences (Waldbieser, personal communication). PCR primers were designed from adjacent exons of selected genes. The intron sequences were amplified and sequenced for the existence of microsatellites. It appeared that this approach is also effective for the development of type I markers in catfish. Among the three approaches, the largest effort is being devoted to the second approach. Our current EST project will sequence 20 000 more channel catfish ESTs and 10 000 blue catfish ESTs. Identification of the same

Copyright © 2003 John Wiley & Sons, Ltd. 

genes from both species will allow identification of eSNPs, many of which will fall within genes of known functions. It is clear that mapping of many known genes to the catfish genome will allow large-scale comparative genome analysis.

### Resource families and linkage maps

Linkage maps of catfish were constructed using both interspecific hybrid resource families [14] and intraspecific channel catfish resource families [34]. Each of the two mapping populations has its own advantages. When using the channel catfish intraspecific resource families, recombination frequency is more natural and thus the genetic distances between markers are more natural. Practical objectives of the map may be toward fine mapping of performance traits showing variation among various strains/lines of channel catfish. A genetic linkage map has been constructed, using the channel catfish resource families. To date, 270 microsatellites have been mapped in 32 linkage groups [34]. By comparison, the use of the interspecific hybrid resource families allows exploitation of an experimental system where maximum polymorphism can be created for markers of various kinds. In this regard, most markers should be species-specific markers and thus data is most likely transferable among different interspecific resource families. Because of the high polymorphism between the channel catfish and blue catfish, the hybrid system should allow mapping of various markers, such as RAPD, AFLP, microsatellites and SNP markers. The practical objectives of using the interspecific hybrid system are to construct synthetic catfish breeds through introgression. While channel catfish are different from, and superior to, blue catfish in growth rate, feed conversion efficiency and resistance to columnaris disease (caused by *Flavobacterium columnare*, the most common bacterial disease in catfish), blue catfish is different from, and superior to, channel catfish in processing yield and in resistance to enteric septicaemia of catfish (ESC, caused by *Edwardsiella ictaluri*, the most severe bacterial disease in catfish). This interspecific system, therefore, provides a model system for analysis of major QTLs involved in disease resistance and disease defences. This system can be exploited for initial mapping, and fine mapping can then be conducted, using either the interspecific or intraspecific mapping populations. A genetic linkage map has been constructed using the interspecific hybrid resource families; 418 AFLP markers have been mapped to 44 linkage groups [14]. Theoretically, the map distance can be lower in the interspecific hybrid system because the homologous recombination rate should be lower between the less homologous sequences of channel catfish and blue catfish.

### QTL mapping and identification of candidate genes

Catfish offer unique advantages for analysis of QTLs. Thousands of individuals can be easily produced per spawn. The use of full-sib families for analysis of quantitative traits should minimize any variation due to use of different families. Large families also allow phenotypic selection. However, labelling fish is often difficult and causes wounding of fish, which interferes with phenotypic evaluations and measurements, e.g. labelling fish, no matter if it is heat branding or pit tagging, often leads to wounding that may stress the fish and results in infections by bacterial pathogens. Several traits are very important for aquaculture, including growth rate, feed conversion efficiency, disease resistance, body conformation and processing yield, tolerance to low dissolved oxygen, and tolerance to low water quality. Feed conversion efficiency is very important because feed accounts for over 50% of variable production cost. Diseases can cause up to 30% of annual losses. Under intensive aquaculture conditions, disease problems have been one of the top concerns of catfish producers.

Selective genotyping is a very effective approach for initial QTL analysis in catfish. Because of large families, selection pressure can be applied for phenotypic evaluations and measurements, such that the phenotypic extremes may be selected for genotyping. For certain markers, such as SNPs, selective genotyping coupled with DNA sample pooling has been shown to be very efficient for initial identification of performance trait linked markers. To date, three markers have been identified as tentatively linked to feed conversion efficiency, and several markers are being evaluated for their linkage with disease resistance to the bacterial disease enteric septicemia of catfish (ESC). QTL projects are ongoing at both Auburn

Copyright © 2003 John Wiley & Sons, Ltd.

University and the USDA ARS Catfish Genetics Research Unit.

### Repetitive elements in the catfish genome and the mitochondrial genome

Understanding of basic genomic composition is very important for decision making related to marker development, linkage mapping and physical mapping. Several repetitive elements have been identified and characterized in catfish. The *Xba* elements are highly repetitive, accounting for about 5% of the catfish genome. They are about 330 bp in size, highly A/T-rich, and arranged in a head-to-tail arrays. These elements appeared to be specific only for channel catfish and blue catfish, but were not in the genomes of several other ictalurid catfishes [20]. The second major class of repetitive elements identified from catfish was *TC1*-like transposons. Several families of *TC1*-like transposon elements have been identified by PCR, using a single primer designed from the inverted repeats. Three of these families have been characterized. The largest *TC1*-like element, referred to as *Tip1*, is 1.6 kb in size, representing the full length *TC1*-like elements; they are highly similar to those identified from zebrafish and other teleosts [2,24,32]. The second family of *TC1*-like elements identified from catfish, referred to as *Tip2*, is 1.0 kb in size, representing the deleted forms of *TC1*-like elements. Sequence comparison of the *Tip2* with known *TC1*-like elements from various organisms indicated that they are more similar to *TC1*-like elements from invertebrates than to those from vertebrates, especially when the functional domains are considered. The third family of *TC1*-like elements consists of non-autonomous *TC1*-like elements, referred to as *Tipnon* [22]. They include inverted repeats that share sequence identity with the *TC1*-like elements, but do not have any sequences homologous to the transposase gene; they are very small, with a size of about 530 bp. However, *Tipnon* is highly abundant, with about 32 000 copies in the catfish genome accounting for about 1.6% of the catfish genome. In addition to the *Xba* elements and the *TC1*-like elements, the *Mermaid* and *Merman*, short interspersed elements (SINE) were also identified and characterized in catfish. About 9000 copies of *Mermaid* and 1200 copies of *Merman* exist in the channel catfish genome. They were so named because of their co-existence [8].

The channel catfish mitochondrial genome has been sequenced and is typical of vertebrate mitochondrial genomes. At least 35 mitochondrial haplotypes have been identified in catfish populations. The sequence and polymorphic information should be useful for parentage analysis and strain analysis in catfish [36].

### Development of genomic resources

Development of genomic resources and technology is one of the recent major focuses in the catfish genomic community. To date, one genomic λ DNA library has been made [8,10] and three BAC large insert DNA libraries have been made ([31]; Pieter DeJong, unpublished). The BAC library made by Dr DeJong as part of a Genome Reagents and Tools Project is widely distributed among the aquaculture genomics community. Its use for physical mapping and other comparative genomic work should enhance information exchange and communication.

Twenty-one cDNA libraries have been made from channel catfish, including 15 cDNA libraries made from various channel catfish tissues and six cDNA libraries made from cultured cell lines. Tissues used for construction of the cDNA libraries include head kidney [1], spleen [11], skin [5], liver, brain [4], stomach, intestine, ovary, gill, muscle, testis, pituitary [6], olfactory tissue, and trunk kidney (posterior kidney); 14 of the 15 cDNA libraries were made in the pSport-1 vector (Life Technologies, MD) and one was made in the λ Unizap cloning vector (Stratagene, CA). In consideration of their uses for the identification of SNPs, tissues from 15 fish were used; as genomic resources to include potentially most, if not all, transcripts for the study of disease-related genes, tissues were collected from both healthy and infected fish at various times after infection. The six cDNA libraries from cultured cell lines of channel catfish were from the catfish autonomous (immortal) B cell line, the catfish autonomous (immortal) T cell line, 1 week-old catfish mixed leukocyte culture, the catfish autonomous (immortal) macrophage cell line, and the catfish non-autonomous (mortal) cytotoxic T cell lines (**http://morag.umsmed.edu/libraries/index.html**).

Six cDNA libraries were also constructed from blue catfish using tissues of head kidney, spleen,

Copyright © 2003 John Wiley & Sons, Ltd. 

liver, gill, skin and heart. These cDNA libraries are being used to conduct comparative analysis of ESTs between the channel catfish and blue catfish for the identification of eSNPs. EST analysis has proven to be one of the most efficient approaches for gene identification, gene expression profiling and cataloguing. It also produces markers and resources for the development of cDNA microarrays [13]. In catfish, over 11 000 ESTs have been sequenced and deposited in GenBank [1,4,5,6,11]. The Institute of Genome Research (TIGR) has constructed a gene index that includes 5905 unique sequences (**http://www.tigr.org**). This number should have been greater, since several more thousands of ESTs have been sequenced recently that have not been deposited to GenBank.

Microarrays are not yet available for catfish, mainly because the numbers of available genes are still small. However, low-density microarrays with 660 genes have been used to study differentially expressed genes during cold temperature stresses and cold acclimation in both channel catfish [3] and white catfish [9]. With the availability of a greater number of ESTs, microarray technology is being used for the identification of differentially expressed genes after disease infection.

### Systematic characterization of genes and/or full length cDNAs

Systematic analysis of complete cDNAs and/or genes using a genomic approach is part of our genome program. While understanding the genome in a large-scale 'forest view' is important, detailed analysis of genes and their expression is mandatory for the understanding of gene structure, gene evolution, gene families, orthologues vs. paralogues, and gene expression in relation to function. Additional information concerning transcript processing can also be obtained regarding alternative splicing and alternative polyadenylation. All such information should facilitate comparative functional genomics. A set of transcripts involved in a specific metabolic pathway or a specific process can be obtained systematically during large-scale EST analysis without screening for specific cDNAs one by one. After initial identification of ESTs representing genes of interest, complete cDNA sequences can be easily obtained and their expression analysed. Using such an approach, we have characterized a complete set of all 32 small ribosomal protein cDNAs [7] and a complete set of all 47 large ribosomal protein cDNAs [30] from channel catfish. Other genes we have analysed include: the myostatin gene [10]; gonadotropin (GnRH) $\alpha$-subunit [17]; GnRH $\beta$-subunit 1 and GnRH $\beta$-subunit 2 [15]; the $\alpha$-actin gene [8]; the creatine kinase gene [16]; a number of cytokine genes; a number of complement genes; and a large number of cytochrome P450 genes (Liu, unpublished).

The significance of characterizing complete coding sequences has been realized; the NIH started its mammalian Full-Length cDNA Initiative in 1999 (**http:grants.nih.gov/grants/guide/rfa-files/RFA-CA-99-005.html**) for the purpose of functional and comparative genomics, and full-length cDNA databases of human (**http://www.ornl.gov/meetings/wccs/helix.htm**) and mouse (**http://www.jsbi.org/journal/GIW99/GIW99P34.pdf**) have also been established in Japan. While our efforts and resources for the analysis of full-length cDNAs are very limited, the high quality cDNA libraries should be a valuable resource for such purposes.

### Bottlenecks and perspectives

Efforts for catfish genome research should be enhanced. Specifically, I believe the following specific areas need to be addressed in the very near future. First, genetic linkage mapping should be continued. Denser genetic maps must be constructed for practical usefulness in breeding programs. QTL mapping efforts should be increased. With the initial identification of performance trait-linked markers, genome regional markers should be developed for the fine mapping of the putative QTLs. Candidate gene identification using microarray and other approaches should be conducted in order to pin down the potential genes involved in important QTLs, especially for disease resistance. Physical mapping using restriction fingerprinting for the construction of BAC contigs should be immediately initiated and funds should be made available for its completion. This is one of the bottlenecks now for catfish genome research. Availability of BAC contigs will allow placement of many known genes onto the physical map through hybridization; it will also allow large-scale comparative mapping and syntenic grouping. More importantly, regional markers can then be developed

Copyright © 2003 John Wiley & Sons, Ltd.

from adjacent BAC clones, for fine mapping of QTLs and for the eventual cloning of economically important genes. In the long term, the BAC contig should be useful as the guide for entire or partial genome sequencing in catfish. Mapping of common markers on the physical map and the linkage maps will also allow integration of various maps that will greatly increase the resolution of catfish maps. Last but not least, comparative mapping efforts should be increased. Much information can be obtained by comparative mapping. The benefits of great investment into basic studies using model species can only be realized by comparative genomics. This includes the 'transfer' of information from map-rich species to catfish and also comparative studies among several aquaculture fish species, such as tilapia and salmonids. A large number of type I markers is being mapped in catfish. Upon completion, comparison of map location of these type I markers can be directly compared to those of the human, cattle, swine and zebrafish. Coordination and mapping of the same set of type I markers in other aquaculture fish species will also allow the development of comparative maps in several other aquaculture species. In addition, hybridization of a common set of type I marker probes to catfish, tilapia and salmonids has been planned (Kocher, personal communication). A direct comparison of their location on the physical map is also possible upon construction of the BAC contigs. Finally, gene expression in relation to function should be studied in a comparative way through evolution. While many genetic mechanisms may have been evolutionarily conserved, specific mechanisms discovered from catfish should fill the gap it represents as an important aquaculture species among the lower teleost fish.

### Acknowledgements

Our catfish genome work has been mostly supported by the USDA NRICGP Animal Genome and Genetic Mechanisms Program, and the USDA NRICGP Animal Genome Basic Reagents and Tools Program, and partially by the Auburn University Competitive Biogrant Program. I am grateful to Dr William Wolters and Dr Rex Dunham for their support and helpful discussions. I thank Dr Geoff Waldbieser and Dr Thomas Kocher for sharing their unpublished information. The author is especially indebted to his past and current dedicated staff and hard working students: Huseyin Kucuktas, Ping Li, Attila Karsi, Chongbo He, Zhenlin Ju, Soohag Kim, Arif Kocabas, Andrea Patterson, Jinian Feng, Micah Simmons, Dongfeng Cao, Kathryn Mickett, Liqiao Chen, Eric Peatman, Putharat Baoprasertkul and Jerry Serapion.

### References

1. Cao D, Kocabas A, Ju Z, *et al*. 2001. Transcriptome of channel catfish (*Ictalurus punctatus*): initial analysis of genes and expression profiles from the head kidney. *Anim Genet* **32**: 169–188.
2. Izsvak Z, Ivics Z, Hackett PB. 1995. Characterization of a *TCI*-like transposable element in zebrafish (*Danio rerio*). *Mol Gen Genet* **247**: 312–322.
3. Ju Z, Dunham R, Liu ZJ. Differential gene expression in the brain of channel catfish (*Ictalurus punctatus*) in response to cold acclimation. *Mol Genet Genom* **268**: 87–95.
4. Ju Z, Karsi A, Kocabas A, *et al*. 2000. Transcriptome analysis of channel catfish (*Ictalurus punctatus*): genes and expression profile from the brain. *Gene* **261**: 373–382.
5. Karsi A, Cao D, Li P, *et al*. 2002. Transcriptome analysis of channel catfish (*Ictalurus punctatus*): initial analysis of gene expression and microsatellite-containing cDNAs in the skin. *Gene* **285**: 157–168.
6. Karsi A, Li P, Dunham R, Liu ZJ. 1998. Transcriptional activities in the pituitaries of channel catfish (*Ictalurus punctatus*) before and after induced ovulation as revealed by expressed sequence tag analysis. *J Mol Endocrinol* **21**: 121–129.
7. Karsi A, Patterson A, Feng J, Liu ZJ. 2002. Translational machinery of channel catfish: I. A transcriptomic approach to the analysis of 32 40S ribosomal protein genes and their expression. *Gene* **291**: 177–186.
8. Kim S, Karsi A, Dunham R, Liu ZJ. 2000. The skeletal muscle $\alpha$-actin gene of channel catfish (*Ictalurus punctatus*) and its association with piscine-specific SINE elements. *Gene* **252**: 173–181.
9. Kocabas A, Dunham R, Liu ZJ. 2003. Differentially expressed genes in the brain of white catfish in response to cold acclimation. *Mol Brain Res* (in review).
10. Kocabas A, Kucuktas H, Dunham RA, Liu ZJ. 2002. Molecular characterization and differential expression of the myostatin gene in channel catfish (*Ictalurus punctatus*). *Biochim Biophys Acta* **1575**: 99–107.
11. Kocabas A, Li P, Cao D, *et al*. 2002. Expression profile of the channel catfish spleen: analysis of genes involved in immune functions. *Mar Biotechnol* **4**: 526–536.
12. Liu Q, Goudi CA, Simco BA, Davis KB, Morizot DC. 1992. Gene-centromere mapping of six enzyme loci in gynogenetic channel catfish. *J Hered* **83**: 245–248.
13. Liu ZJ, Karsi A, Dunham RA. 1999. Development of polymorphic EST markers suitable for genetic linkage mapping of catfish. *Mar Biotechnol* **1**: 437–447.
14. Liu ZJ, Karsi A, Li P, Cao D, Dunham R. 2003. An AFLP-based genetic linkage map of channel catfish (*Ictalurus punctatus*) constructed by using an interspecific hybrid resource family. *Genetics* (in press).
15. Liu ZJ, Kim S, Karsi A. 2001. Channel catfish follicle stimulating hormone and luteinizing hormone: cDNA cloning and their expression during ovulation. *Mar Biotechnol* **3**: 590–599.

Copyright © 2003 John Wiley & Sons, Ltd.

16. Liu ZJ, Kim S, Kucuktas H, Karsi A. 2001. Multiple isoforms and an unusual cathodic isoform of creatine kinase from channel catfish (*Ictalurus punctatus*). *Gene* **275**: 207–215.
17. Liu ZJ, Li P, Argue B, Dunham R. 1997. Gonadotropin $\alpha$ subunit glycoprotein from channel catfish (*Ictalurus punctatus*) and its expression during hormone-induced ovulation. *Mol Mar Biol Biotechnol* **6**: 221–231.
18. Liu ZJ, Li P, Argue B, Dunham R. 1998. Inheritance of RAPD markers in channel catfish (*Ictalurus punctatus*), blue catfish (*I. furcatus*) and their $F_1$, $F_2$ and backcross hybrids. *Anim Genet* **29**: 58–62.
19. Liu ZJ, Li P, Argue BJ, Dunham RA. 1999. Random amplified polymorphic DNA markers: usefulness for gene mapping and analysis of genetic variation of catfish. *Aquaculture* **174**: 59–68.
20. Liu ZJ, Li P, Dunham R. 1998. Characterization of an A/T-rich family of sequences from the channel catfish (*Ictalurus punctatus*). *Mol Mar Biol Biotechnol* **7**: 232–239.
21. Liu ZJ, Li P, Kocabas A, *et al.* 2001. Microsatellite-containing genes from the channel catfish brain: evidence of trinucleotide repeat expansion in the coding region of nucleotide excision repair gene RAD23B. *Biochem Biophys Res Comm* **289**: 317–324.
22. Liu ZJ, Li P, Kucuktas H, Dunham RA. 1999. Characterization of nonautonomous *TC1*-like transposable elements of channel catfish (*Ictalurus punctatus*). *Fish Physiol Biochem* **21**: 65–72.
23. Liu ZJ, Li P, Kucuktas H, *et al.* 1999. Development of AFLP markers for genetic linkage mapping analysis using channel catfish and blue catfish interspecific hybrids. *Trans Am Fish Soc* **128**: 317–327.
24. Liu ZJ, Li P. 2003. Multiple *TC1*-like transposon families from channel catfish (*Ictalurus punctatus*). *Genetica* (in review).
25. Liu ZJ, Nichols A, Li P, Dunham R. 1998. Inheritance and usefulness of AFLP markers in channel catfish (*Ictalurus punctatus*), blue catfish (*I. furcatus*) and their $F_1$, $F_2$ and backcross hybrids. *Mol Gen Genet* **258**: 260–268.
26. Liu ZJ, Tan G, Kucuktas H, *et al.* 1999. High levels of conservation at microsatellite loci among Ictalurid catfishes. *J Hered* **90**: 307–312.
27. Liu ZJ, Tan G, Li P, Dunham RA. 1999. Transcribed dinucleotide microsatellites and their associated genes from channel catfish, *Ictalurus punctatus*. *Biochem Biophys Res Commun* **259**: 190–194.
28. Mickett K, Morton C, Feng J, *et al.* 2003. Assessing genetic diversity of domestic populations of channel catfish (*Ictalurus punctatus*) in Alabama using AFLP markers. *Aquaculture* (in review).
29. Morizot D, Schmidt M, Carmichael G. 1994. Joint segregation of allozymes in catfish genetic crosses: designation of *Ictalurus punctatus* linkage group I. *Trans Am Fish Soc* **123**: 22–27.
30. Patterson A, Karsi A, Feng J, Liu ZJ. 2003. Translational machinery of channel catfish: II. Complementary DNA and expression of the complete set of 47 60S ribosomal proteins. *Gene* (in press).
31. Quiniou SMA, Katagiri T, Clem W, Wolters WR, Waldbieser GC. 2003. Construction and characterization of two BAC libraries from gynogenetic channel catfish, *Ictalurus punctatus*. *Genet Select Evol* (submitted).
32. Radice AD, Bugaj B, Fitch DH, Emmons SW. 1994. Widespread occurrence of the *TC1* transposon family: *TC1*-like transposons from teleost fish. *Mol Gen Genet* **244**: 606–612.
33. Tan G, Karsi A, Li P, *et al.* 1999. Polymorphic microsatellite markers in *Ictalurus punctatus* and related catfish species. *Mol Ecol* **8**: 1758–1760.
34. Waldbieser GC, Bosworth BG, Nonneman DJ, Wolters WR. 2001. A microsatellite-based genetic linkage map for channel catfish, *Ictalurus punctatus*. *Genetics* **158**: 727–734.
35. Waldbieser GC, Bosworth BG. 1997. Cloning and characterization of microsatellite loci in channel catfish, *Ictalurus punctatus*. *Anim Genet* **28**: 295–298.
36. Waldbieser GC, Bilodeau AL, Nonneman DJ. 2003. Complete sequence and characterization of the channel catfish mitochondrial genome. *DNA Sequence* (submitted).

Copyright © 2003 John Wiley & Sons, Ltd. 
